# Supplementary material for: Patterns of Intron Gain and Loss in Fungi
Source: PLoS Biol. 2004 Nov 30;2(12):e422. doi: 10.1371/journal.pbio.0020422 (PMC532390; doi:10.1371/journal.pbio.0020422)
Supplement: Table S1 — Also available at http://genes.mit.edu/NielsenEtAl/. (4.3 MB ZIP). [file pbio.0020422.st001.zip › NielsenEtAl/html/946.html]

AN6992.1.NCU05544.1.MG10055.1.FG08277.1


```
 CLUSTAL W (1.82) Multiple Sequence Alignments - Introns Inserted


Sequence 1: NCU05544.1	92 aa
Sequence 2: MG10055.1	92 aa
Sequence 3: FG08277.1	92 aa
Sequence 4: AN6992.1	92 aa
Alignment Length: 92 aa
Number Identitical Residues: 48 aa
Alignment Score (without introns) 2291


MG10055.1 	MTRAQQTISILLLATS0LYLCLYLQLVPLPDVIQTEVIPV0LPFWVLVSFGALLLGRLGW
NCU05544.1	MTRAQQTISIALLVSS0LYLSLYLELVPLPALVQTAIVPV0LPFWALVSFGAWVLFRLGW
FG08277.1 	MTRAQQTISLALLVSS0LYLALFLELIPLPPLIQEQIVPV0LPFWALVSFGAYLLFRLGF
AN6992.1  	MTRAQQTLSVLLLVSS0FYLSLYLGLIPLNETIQQEIIPV0LPFYALISFGCYLLGRLGL
          	*******:*: **.:* :**.*:* *:**   :*  ::** ***:.*:***. :* *** 

MG10055.1 	GVLTFNDTPEAHKELLVEIEEAKKDLRRLGVTVD
NCU05544.1	GMLTFRDTPEAYKELMEEIKLAQADLRAKGVSVD
FG08277.1 	GILTFNDVPNAHKELTAEIEQAKVELRKLGVTVD
AN6992.1  	AIFTFHDVPEAHKELQGEIEQAKAELRRANVDVD
          	.::**.*.*:*:***  **: *: :**  .* **
```
